# Barrier screens: a method to sample blood-fed and host-seeking exophilic mosquitoes

**DOI:** 10.1186/1475-2875-12-49

**Published:** 2013-02-05

**Authors:** Thomas R Burkot, Tanya L Russell, Lisa J Reimer, Hugo Bugoro, Nigel W Beebe, Robert D Cooper, Supraman Sukawati, Frank H Collins, Neil F Lobo

**Affiliations:** 1 James Cook University, Queensland Tropical Health Alliance, Cairns, QLD, 4870, Australia; 2 Centers for Disease Control and Prevention, Division of Parasitic Diseases and Malaria, Atlanta, GA, 30333, USA; 3 Papua New Guinea Institute of Medical Research, Madang, Papua New Guinea; 4 Case Western Reserve University, Cleveland, OH, 44106, USA; 5 National Vector Borne Disease Control Programme, Ministry of Health, Honiara, Solomon Islands; 6 School of Biological Sciences, University of Queensland, St. Lucia, QLD, 4068, Australia; 7 CSIRO Ecosystem Sciences, Dutton Park, Brisbane, QLD, 4102, Australia; 8 Australian Army Malaria Institute, Gallipoli Barracks, Enoggera, QLD, 4051, Australia; 9 National Institute of Health, Research and Development, Health Ecology Research and Development Center, Jakarta, Indonesia; 10 Eck Institute for Global Health, Department of Biological Sciences, University of Notre Dame, Notre Dame, IN, 46556, USA

**Keywords:** Mosquito sampling, Human blood index, Barrier screen trap, Exophily

## Abstract

**Background:**

Determining the proportion of blood meals on humans by outdoor-feeding and resting mosquitoes is challenging. This is largely due to the difficulty of finding an adequate and unbiased sample of resting, engorged mosquitoes to enable the identification of host blood meal sources. This is particularly difficult in the south-west Pacific countries of Indonesia, the Solomon Islands and Papua New Guinea where thick vegetation constitutes the primary resting sites for the exophilic mosquitoes that are the primary malaria and filariasis vectors.

**Methods:**

Barrier screens of shade-cloth netting attached to bamboo poles were constructed between villages and likely areas where mosquitoes might seek blood meals or rest. Flying mosquitoes, obstructed by the barrier screens, would temporarily stop and could then be captured by aspiration at hourly intervals throughout the night.

**Results:**

In the three countries where this method was evaluated, blood-fed females of *Anopheles farauti*, *Anopheles bancroftii, Anopheles longirostris, Anopheles sundaicus*, *Anopheles vagus*, *Anopheles kochi, Anopheles annularis*, *Anopheles tessellatus, Culex vishnui*, *Culex quinquefasciatus* and *Mansonia* spp were collected while resting on the barrier screens. In addition, female *Anopheles punctulatus* and *Armigeres* spp as well as male *An. farauti*, *Cx. vishnui*, *Cx. quinquefasciatus* and *Aedes* species were similarly captured.

**Conclusions:**

Building barrier screens as temporary resting sites in areas where mosquitoes were likely to fly was an extremely time-effective method for collecting an unbiased representative sample of engorged mosquitoes for determining the human blood index.

## Background

The importance of a mosquito species as a vector of human disease is determined by multiple parameters including the human blood index (HBI). The HBI is the proportion of blood-fed, resting mosquitoes that contain human blood in their stomachs compared to the total number of mosquitoes which feed on any host species [1]. Accurate estimates of the HBI for a species depends on collecting an unbiased sample of resting, blood-fed mosquitoes of that species [2]. For highly endophilic species that rest for prolonged periods inside houses, it is relatively easy and straightforward to collect large numbers of engorged mosquitoes inside houses. However, HBI estimates based solely on collections of indoor-resting mosquitoes, even for endophilic mosquitoes, are biased towards human-fed mosquitoes, as such collections ignore the portion of the population that will have fed outdoors and be more likely to have fed on other available host species. Accurate HBI estimates require unbiased samples of the entire mosquito population that has recently fed (i.e., those that feed and rest inside houses as well as those that feed and rest outside).

Exophilic mosquito species are common throughout the malaria-endemic world [3-5]. For exophilic vectors, capturing an adequate and representative sample of blood-fed specimens is even more challenging as they tend to be dispersed over large areas and utilize a large number of potential resting sites. The challenge is magnified when the number of adult vectors is limited, as finding engorged resting mosquitoes outdoors requires considerable time and effort to acquire even a small sample of blood-fed mosquitoes [2]. To enhance the prospects of finding blood-fed mosquitoes outside houses, artificial resting sites, such as clay pots and resting boxes may be provided or pits dug to attract engorged females [6]. However, artificial resting sites may harbour relatively few blood-fed, resting mosquitoes since the artificial sites provided must compete with the greater number of available natural resting sites [7,8].

In response to biases associated with sampling blood-fed, resting mosquitoes, a novel sampling tool was designed based on a hypothesis that engorged mosquitoes might be intercepted and captured when transiting between blood feeding and resting sites. This hypothesis was based on observations by Giglioli [9] and Gillies and Wilkes [10] about the flight patterns of anophelines. Giglioli [9] reported that *Anopheles melas* entered villages in corridors and at altitudes less than five feet (1.53 m) and that their flight could be diverted by fences. Gillies and Wilkes [10] confirmed that most mosquitoes fly at low altitudes when crossing open terrain although anophelines will modify the height of their flight when they encounter obstacles. It followed that obstacles could be constructed, not to divert flying mosquitoes, but to impede their flight sufficiently to allow capture. For blood-laden mosquitoes, any structure encountered between the host and sites for resting might be sufficient to provide a temporary rest stop for the mosquito. Barrier screens constructed of durable mesh material (shade cloth) are inexpensive, easily constructed, and easily searched for resting mosquitoes from which they can be collected. As an artificially constructed resting site or flight barrier, a screen provides an additional significant advantage over a solid structure in that the mesh is permeable and would permit mosquitoes to follow both visual and olfactory cues to sources of blood meals, oviposition and resting sites. Upon encountering the barrier screen, mosquitoes might then stop, thereby facilitating their discovery and capture. Placement of a barrier screen between likely oviposition/resting sites and potential blood meal sources would enable the capture of both blood-fed mosquitoes seeking a resting site to develop their eggs, as well as recently emerged mosquitoes that have completed egg-laying and/or are searching for a blood meal. Comparisons of blood meal, parity and gravid status of mosquitoes captured on each side of such a screen would provide insight into directional and temporal behaviour patterns.

This initial evaluation of barrier screens as a novel sampling tool to collect exophilic mosquitoes was conducted in three countries in the southwest Pacific (Indonesia, the Solomon Islands and Papua New Guinea). The current paper presents the results of pilot studies to optimise the use and placement of barrier screens and to describe the physiological state of the mosquitoes captured. In this region the primary malaria and lymphatic filariasis vectors are exophilic, including *An. sundaicus*, *An. vagus*, *An. kochi, An.s annularis, An. tessellatus* and the members of the *An. punctulatus* group [3,11,12]. Previous HBI estimates for the *An. punctulatus* group required years of effort to collect a sufficient number of blood-fed, resting specimens for blood source identification. This was primarily due to the difficulty of finding mosquitoes resting amongst the thick vegetation that serve as the usual resting sites for these mosquitoes [6,13]. Thus, only a limited number of studies have documented the host blood meal sources in the members of this group in the Solomon Islands [14-16] and Papua New Guinea [13,16-22], the most recent of which was published 16 years ago.

## Methods

### Barrier screens

**Figure 1:**
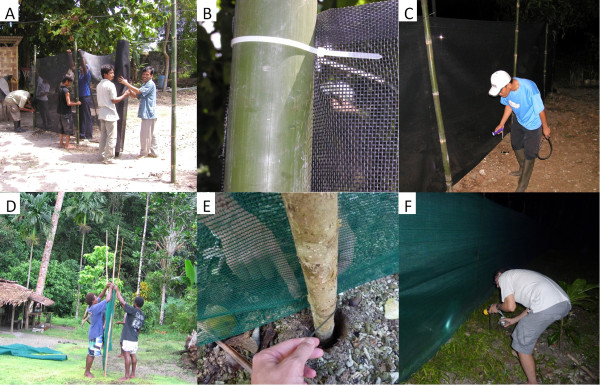
**Construction and use of 2 m high barrier screens for sampling mosquitoes in Indonesia (A, B, C) and the Solomon Islands (D, E, F).** In Indonesia, shade cloth **(A)** was attached to bamboo poles with zip-ties **(B).** In the Solomon Islands, barrier screens were made with polyethylene shade cloth **(D)** attached to bamboo poles with polyester cord **(E).** Screens were searched by flashlight and resting mosquitoes captured by aspiration **(C**, **F)**.

The barrier screens were constructed from ether polyvinylchloride-coated polyester or polyethylene shade cloth (70% shading) netting. The shade cloth was obtained either from the manufacturer [23] or local hardware stores. The 2 m-high barrier screens were constructed by securing the shade cloth to wooden or bamboo poles at 2 m intervals (Figure 1A,D) with zip-ties or polyester cord (Figure 1B,E, respectively) and the barrier screens searched during the night for resting mosquitoes, which were then captured by aspirations (Figure 1C,F). The efficacy of the barrier screens for facilitating the collection of exophilic mosquitoes was evaluated in sites in three countries.

### Mosquito sampling

The experiments involved sampling mosquitoes from the barrier screens or by using human landing catch (HLC) in all three sites. All mosquito catches were conducted between 1800 h and 0600 h, and the catches for each hourly interval were stored in separate collection cups. Regarding sampling resting mosquitoes from the barrier screens, this process was conducted by manually searching the barrier screen and collecting any resting mosquitoes with a mouth aspirator (Figure 1C and F). Each side of the barrier screen was searched for approximately 20mins each hour. The catches for each side of the barrier screen were stored in separate collection cups. To collect host-seeking mosquitoes HLC were conducted outdoors. This involved volunteers sitting with their legs and feet exposed and catching mosquitoes with a mouth aspirator that were attracted and seeking a blood meal; HLC collections were also made hourly from 1800 h to 0600 h [6]. All mosquitoes were morphologically identified to sex and species then visually classified as being unfed, partially fed, fully fed or gravid. The morphological keys used to identify the mosquito specimens were O'Connor and Soepanto [24] in Indonesia, Belkin [11] in the Solomon Islands, and Lee [25] in Papua New Guinea. Species identifications were confirmed by molecular analyses (see Laboratory analyses below). The collection details for each mosquito were recorded, including trap type and hour of capture.

### Indonesia

A 32 m long barrier screen of polyvinylchloride-coated polyester shade cloth was constructed (Figure 1A,B) and evaluated in Sukaraja village in the Lampung District (Rajabasa subdistrict) of southern Sumatra in western Indonesia between 25 August and 1 September 2010. Mosquitoes were collected from the barrier screen over eight nights. The location of mosquitoes was recorded for each mosquito (e.g., <0.5 m; 0.5 to <1.0 m; >1 m above the ground). Domestic animals that might serve as potential host blood meal sources were recorded. After the mosquito processing was complete, unfed female anophelines were dissected for parity status. Additionally, HLC was performed on the same nights as the barrier screen collections at 6 outdoor stations.

### Solomon Islands

**Figure 2:**
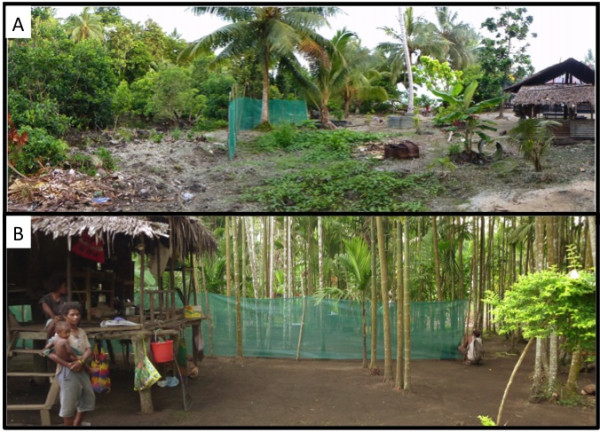
**Barrier screens were constructed between village houses and potential resting and/or oviposition sites, as shown in Haleta village, Solomon Islands (A) and Mirap village, Papua New Guinea (B).** Potential resting sites among the vegetation and the primary oviposition site (a brackish water swamp) can be seen to the right of the barrier screen while village houses and animal pens (seen to the left of the barrier screen) provide potential blood meals.

In Haleta village on Big Nggela Island, Central Province, Solomon Islands, the barrier screen experiments were refined to intercept mosquitoes either seeking blood meals or searching for resting sites. The layout of Haleta village provided an ideal scenario to test a hypothesis that barrier screens could intercept mosquitoes entering the village searching for a blood meal after having just emerged or after having just laid eggs, as well as intercepting blood-fed mosquitoes leaving the village and seeking a resting site to develop their eggs. Previously, larval surveys identified a single dominant breeding site, a swamp formed by the blockage of a stream by a sandbar. This swamp was surrounded by thick vegetation, suitable resting sites for blood-fed mosquitoes, whereas in the village where there was little or no vegetation that might serve as resting sites. A barrier screen of approximately 20 m was constructed between the swamp and the village houses (Figure 2A). Resting mosquitoes were collected from the barrier screen for 14 consecutive nights in November 2011.

Concurrent with barrier screen collections, HLC were conducted from 1800 h to 0600 h at 6 outdoor stations. Mosquitoes were held in individual containers by hour of collection and numbers recorded.

### Papua New Guinea

A 40 m barrier screen was similarly constructed between houses and the surrounding vegetation in Mirap village, Madang Province (Figure 2B). The barrier screen was constructed in two 20-m segments for ease of set-up and to allow a 1 m gap for access to the surrounding bush along pre-existing trails. One pair of collectors checked the barrier screen for resting mosquitoes for 10 nights in June 2012. The mosquitoes were captured and stored by hour of collection and side of the barrier screen. Additionally, HLC was performed on the same nights as the barrier screen collections at 2 outdoor stations. Locations of screens and collectors were rotated throughout the village every two nights.

### Laboratory analyses

Mosquito species identification of the *An. punctulatus* group was confirmed by PCR [26,27]. Sequencing of the ITS2 region [26] was also used to identify species in Indonesia. Host blood meal sources (human, pig, goat, and dog) for *Anopheles* mosquitoes were identified by PCR using slight modifications of the Kent and Norris method [28]. The PCR reactions were made up of 0.4 μl of each primer, 3.0 mM MgCl_2_, 1.0 mM dNTPs and 0.5 units of Taq polymerase and each reaction in a final volume of 25 μl.

### Statistical analyses

For the Solomon Islands, statistical differences in the proportion of blood-fed to unfed mosquitoes resting on each side of the barrier screen were compared using a generalized linear model (GLM) with a binomial distribution and a categorical explanatory variable for screen side. The basis of the analysis was a binary dataset constructed with the total number of fed and unfed *An. farauti* collected on each side of the barrier screen by date. This analysis was conducted using R statistical software (ver.2.14.2).

### Ethics

Ethical approval for the study was obtained from review boards relevant to each study site. For Indonesia approval was granted by the National Institute of Health, Research and Development, Indonesia. For the Solomon Islands, approval was granted by the National Health Research & Ethics Committee (02-05-2011) as well as the James Cook University Human Research Ethics Committee (H4122). For Papua New Guinea, approval was granted by the PNG Institute of Medical Research Institutional Review Board (1116) and the PNG Medical Research Advisory Board (11.21). When the study commenced, permission was obtained from each person who volunteered to conduct HLC. After consenting, each volunteer signed an informed consent form stating their willingness to participate in the study.

## Results

### Indonesia

In southern Sumatra, *Cx. vishnui* and *Cx. quinquefasciatus* were the most abundantly collected species on the barrier screen (n= 1,057 and 513, respectively), including 65 and 117 male *Cx. vishnui* and *Cx. quinquefasciatus,* respectively. Five species of anophelines were collected resting on the barrier screen. *Anopheles sundaicus* (n= 26) was the most abundantly collected anopheline followed by *An. vagus* (n= 15), *An. kochi* (n= 2) and *An. annularis* and *An. tessellatus* (one each) (Table 1). The mean number of *An. sundiacus* collected per 10 m barrier screen per night was 1.0 and for *An. vagus* was 0.6. The mean nightly, human landing rate for *An. sundiacus* during the same period was 7.6 bites/person/night (b/p/n) and for *An. vagus* was 0.3 b/p/n. The host seeking densities of *An. tesselatus*, *An. kochi* and *An. annularis* were negligible (Table 1).

**Table 1:** Tabular comparison of the density of host-seeking (HLC) and resting mosquitoes (barrier screen) caught in the three study sites: Indonesia, the Solomon Islands and Papua New Guinea

	**Host seeking**	**Resting mosquitoes**				
	**HLC b/p/n (total)**	**Mean/10 m barrier screen (total)**		**Abdominal status**		
**Species**	**Female**	**Male**	**Female**	**Unfed% (n)**	**Bloodfed% (n)**	**Gravid% (n)**
***INDONESIA***						
*An. sundiacus*	7.6 (367)	0.0 (0)	1.0 (26)	58.3 (14)	25.0 (6)	16.7 (4)
*An. vagus*	0.3 (15)	0.0 (0)	0.6 (15)	53.5 (8)	26.7 (4)	20.0 (3)
*An. tesselatus*	0.0 (0)	0.0 (0)	0.0 (1)	100.0 (1)	0.0 (0)	0.0 (0)
*An. kochi*	0.0 (2)	0.0 (0)	0.1 (2)	50.0 (1)	50.0 (1)	0.0 (0)
*An. annularis*	0.0 (1)	0.0 (0)	0.0 (1)	100.0 (1)	0.0 (0)	0.0 (0)
*Cx. quinquefasciatus*	NA	4.2 (117)	15.5 (396)	85.6 (338)	5.6 (22)	8.9 (35)
*Cx. vishnui*	NA	2.5 (65)	38.8 (992)	91.4 (907)	6.5 (64)	2.1 (21)
***SOLOMON ISLANDS***						
*An. farauti s.s.*	16.5 (1388)	0.1 (1)	4.2 (117)	36.8 (43)	62.4 (73)	0.9 (1)
***PAPUA NEW GUINEA***						
*An. bancrofti*	0.0 (0)	NA	0.8 (30)	76.7 (23)	23.3 (7)	0.0 (0)
*An. farauti s.s.*	20.6 (412)	NA	7.8 (311)	40.2 (125)	59.2 (184)	0.6 (2)
*An. punctulatus*	0.0 (0)	NA	0.1 (3)	100.0 (3)	0.0 (0)	0.0 (0)
*An. longirostris*	0.0 (0)	NA	0.1 (2)	50.0 (1)	50.0 (1)	0.0 (0)
*Aedes* spp.	NA	NA	1.4 (54)	79.6 (43)	20.4 (11)	0.0 (0)
*Culex* spp.	NA	NA	1.1 (42)	90.5 (38)	7.1 (3)	2.4 (1)
*Mansonia* spp.	NA	NA	0.1 (2)	50.0 (1)	50.0 (1)	0.0 (0)

The abdominal status of resting mosquitoes is noted.

b/p/n = bites/person/night; NA = Not Available.

When capturing mosquitoes on the barrier screen, it was observed that most (92%) were captured resting <1 m from the ground, and of these mosquitoes 74% were within 50 cm of the ground. All anophelines were collected <1 m from the ground with 73% within 50 cm of the ground. Of the anophelines resting on the barrier screen, 25.5% (11/43) were blood fed, whereas only 6.5% (64/992) and 5.6% (22/395) of *Cx. vishnui* and *Cx. quinquefasciatus* were blood fed. Gravid females represented 16.3% (7/43), 2.1% (21/992) and 8.9% (35/395) of anophelines, *Cx. vishnui* and *Cx. quinquefasciatus* collected on the barrier screen, respectively.

Goats, chickens, humans, dogs and cats were observed in the vicinity of the barrier screen. Only a small portion of the engorged females were tested for blood meal with PCR. All tested *An. sundiacus* contained dog blood (n= 2) while all tested *An. vagus* had fed on goats (n= 3). For *Cx. quinquefasciatus*, 25%, 50% and 25% of identified blood meals were on goats, dogs and humans, respectively (n= 8 successful PCR reactions where the blood meal host was identified, 5 additional blood meal tests could not be identified). For *Cx. vishnui*, 59%, 20.5% and 20.5% of identified blood meals were on goats, dogs and humans, respectively (n= 49 successful PCR reactions, 3 could not be identified). The parity rate of the *Anopheles* spp was high at 86% (24/28) and also for the *Culex* spp at 71% (89/125).

### Solomon Islands

**Figure 3:**
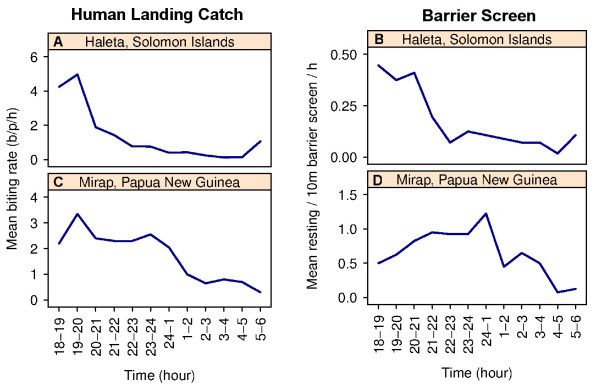
**Comparison of the mean densities of *Anopheles farauti *captured with human landing catches and resting on the barrier screen in the Solomon Islands (A, B, respectively) and Papua New Guinea (C, D, respectively).**

A total of 117 female *An. farauti* were collected on the barrier screen (mean of 4.2/10 m barrier screen/per night; Table 1) with 84% (n= 98) captured on the village side and only 16% (n= 19) on the side closest to the oviposition and resting sites. In addition, seven male *Aedes* spp as well as one *Culex* spp and one *An. farauti* male were collected on the barrier screen. Six of the seven male *Aedes* spp were captured between 0200 h and 0500 h. The number of resting *An. farauti* peaked between 2000 h and 2100 h and then diminished and remained low for the remainder of the night (Figure 3B). During the 14 nights of the experiments, the mean nightly outdoor landing catch for *An. farauti* was 16.5/p/n (Table 1). Peak human landing catches occurred between 1900 h and 2000 h (Figure 3A).

The percentage of blood-fed female *An. farauti* captured resting on the barrier screen was 62% (n= 73), with 51% (n= 60) of the total mosquitoes being fully engorged. There was a tendency for more blood-fed *An. farauti* to be captured on the village side of the barrier screen (65%) compared with the breeding-site side (47%), but this difference was not statistically significant (*β*=0.632, SE=0.517, *p*=0.221). Only one gravid *An. farauti* was collected on the barrier screen. The most abundant potential domestic host blood meal sources present in the village in the vicinity of the barrier screen were humans (n= 64), pigs (n= 11) and dogs, (n= 4). The majority, 97% (n= 68), of blood-fed *An. farauti* contained human blood including 6% (n= 4) having mixed feeds on humans and pigs. Only 3% (n= 2) of *An. farauti* had fed solely on pigs.

### Papua New Guinea

A total of 449 female mosquitoes were collected from the barrier screen with 50% from the village side. Across all species, of those collected from the village side, 54% were blood fed (n= 144) whereas only 34% were blood fed (n= 63) on the opposite side (furthest from the village). *Anopheles farauti* was the predominant species, comprising 69% of the catch (n= 311). In addition, *Anopheles bancroftii* (n= 30), *Anopheles longirostris* (n= 2), *An. punctulatus* (n= 3), *Aedes* spp (n= 54), *Culex* spp (n= 42), *Mansonia* spp (n= 2) and *Armigeres* spp (n= 5) were collected (Table 1).

The mean number of *An. farauti* collected per 10 m barrier screen per night was 7.8 (Table 1). By abdominal status these *An. farauti* were: 59.2% blood fed (n= 184), 40.2% unfed (n= 125) and 0.6% gravid (n= 2) (Table 1). The mean nightly, human landing rate for *An. farauti* during the same period was 20.6/p/n (n= 412) with the major landing collection peak occurring between 1900 h and 2000 h and a secondary peak between 2300 h and midnight (Figure 3C). Peak collection time for resting mosquitoes on the barrier screen was between midnight and 0100 h (Figure 3D). *Anopheles bancroftii*, *An. longirostris* and *An. punctulatus* were not captured in landing catches during the nights when collections on the barrier screens were undertaken.

## Discussion

The hypothesis that a barrier screen trap can detain mosquitoes sufficiently to allow their collection was validated in all three countries where trialled. The barrier screen was specifically designed for collecting an unbiased sample of outdoor resting mosquitoes to provide specimens for calculating the human blood index. This novel tool could directly replace unproductive and/or biased searches for mosquitoes resting outdoors in vegetation, indoors or in outdoor shelters. The use of barrier screens was more time-effective as a means of collecting resting, blood-engorged members of the *An. punctulatus* group, when population densities are low, compared to searching natural resting sites. Previous studies in Mebat village, PNG, in which the bush was searched for resting anophelines, found 27 engorged members of the *An. punctulatus* group during 128 days of searching when the mean nightly human landing rate was 8/p/n [18]. In the same study, only 12 engorged anophelines were collected in Hudini village during 128 search days despite a mean nightly human landing rate of 85/p/n. The use of a barrier screen as a sampling tool for resting mosquitoes was more time effective: in the Solomon Islands over a 14-day period, 70 blood-fed *An. farauti* were collected even though the human landing rate during this period was only 16.5/p/n; in PNG, 184 engorged *An*. *farauti* were captured on the barrier screen in a 10-day period when the human landing catch averaged 20.6/p/n.

Positioning the barrier screen between the hosts and oviposition/resting sites enabled samples of blood-fed mosquitoes to be collected to estimate the HBI as well as to sample mosquitoes questing for blood meals. In Indonesia analyses of the limited numbers of blood meals in anophelines confirmed previous descriptions of *An. vagus* and *An. sundaicus* as zoophilic [3] as human blood was not identified in any anopheline collected on the barrier screen. Although only a very limited number of blood-fed anophelines were collected in Indonesia, the results were sufficiently encouraging to continue the experiments in the Solomon Islands and PNG. Blood meal analyses were conducted on *An. farauti* collected in the Solomon Islands, and at this study site the HBI was very high. Previous literature indicated that *An. farauti* blood-feeding habits vary from zoophilic to anthropophilic [13,16-22,29]. The high HBI recorded in this study may be a function of the relative abundance of humans compared to domestic animals (human, pig, and dog populations in the vicinity of the barrier screen numbered 63, 11 and 4, respectively) and/or an innate preference for feeding on humans.

The density of *An. farauti* captured on the barrier screen paralleled the results of human landing catches with most resting *An. farauti* captured between 1800 h and 2100 h in the Solomon Islands and before midnight in PNG. These results, coupled with anecdotal observations made during the course of the experiments indicate that the barrier screen does indeed act as a barrier which temporarily detains mosquitoes, opposed to being an artificial resting site. Notably, greater numbers of resting *An. farauti* captured on the village side of the barrier screen may reflect the willingness of blood-fed *An. farauti* to rest longer on the barrier screen than mosquitoes questing for blood. Such questing mosquitoes would be more likely to encounter the barrier screen on the oviposition site side but would be expected to fly over or around the barrier screen to continue following cues to the locations of potential blood meals in the village. The success of the barrier screen as a tool for collecting blood-fed mosquitoes provides the baseline for designing detailed experiments to systematically observe individual mosquito resting behaviour as well as to study the population-level feeding behaviour. Of note here that is the observation that most mosquitoes, and particularly anophelines, rest near the ground is consistent with both the observations in The Gambia of Giglioli [9] for *Anopheles melas* and Damar *et al.*[30] in Indonesia who reported that *Anopheles aconitus*, *Anopheles subpictus* and *Anopheles indefinitus* rest indoors at a median height of 38 cm above the floor.

Although the experiments were designed to sample female anophelines, the barrier screen also useful for sampling culicine species, with >1,500 specimens of two *Culex* species being captured during seven nights in Indonesia. In addition, a small number of male *Anopheles* and *Aedes* spp were also collected on the barrier screens. Interestingly, most male *Aedes* were captured on the barrier screen between 0200 h and 0500 h. With further refinements, screens or similar barriers may provide a simple method for sampling the male mosquito population, an area in which far too little is presently known.

The potential use of novel barrier screens as a method for sampling blood-fed mosquitoes seeking resting sites, female mosquitoes questing for blood meals and male mosquitoes, was evaluated in three countries that are well known for their exophilic and exophagic anophelines. Importantly, the technique is largely free of the biases associated with solely collecting mosquitoes resting inside households [2] and is much more effective than searching for outdoor-resting mosquitoes in the natural vegetation. The approach was validated as a means of sampling engorged females for HBI determination and shows promise as an approach to sampling host-seeking female mosquitoes as well as male mosquito populations. It is proposed that barrier screens could replace direct searching of vegetation for collecting outdoor-resting anophelines in many locations.

## Conclusions

Barrier screens placed to intercept mosquitoes between blood feeding, resting and ovipositing provide a novel way to sample mosquitoes. These barrier screens are easy and economical to construct and are effective in capturing *Anopheles*, *Culex* and *Aedes* spp including both males and females. Manipulating the locations where barrier screens are placed provides opportunities for capturing mosquitoes to better understand when and where mosquitoes move between host-seeking, egg laying and resting activities.

## Competing interests

The authors declare that they have no competing interests.

## Authors’ contributions

TRB conceived the concept and instituted the initial experimental designs. TLR, LJR and SS supervised the overall field studies in the Solomon Islands, PNG and Indonesia, respectively. HB, NWB and RDC contributed to the experimental designs and conducted the field studies in the Solomon Islands. FHC and NFL contributed to the experimental designs and coordinated the studies in the Solomon Islands and Indonesia countries. All authors read and approved the final manuscript.
